# Exposing Obstetric Violence in the Eastern Mediterranean Region: A Review of Women's Narratives of Disrespect and Abuse in Childbirth

**DOI:** 10.3389/fgwh.2022.850796

**Published:** 2022-04-25

**Authors:** Merette Khalil, Kashi Barbara Carasso, Tamar Kabakian-Khasholian

**Affiliations:** ^1^Your Egyptian Doula, Cairo, Egypt; ^2^International Course for Health and Development, Health Unit, KIT Royal Tropical Institute, Amsterdam, Netherlands; ^3^Department of Health Promotion and Community Health, Faculty of Health Sciences, American University of Beirut, Beirut, Lebanon

**Keywords:** childbirth, respectful maternity care (RMC), obstetric violence, Eastern Mediterranean, SRHR, disrespect and abuse (D&A), women-centered care

## Abstract

**Background:**

Obstetric violence (OV) threatens the provision of dignified, rights-based, high-quality, and respectful maternal care (RMC). The dearth of evidence on OV in the Eastern Mediterranean Region poses a knowledge gap requiring research to improve rights-based and respectful health practice and policy. While efforts to improve the quality of maternal health have long-existed, women's experiences of childbirth and perceptions of dignity and respect are not adequately or systematically recorded, especially in the said region.

**Aim:**

This study centered on the experiences of women's mistreatment in childbirth to provide an overview of OV and offer recommendations to improve RMC.

**Methods:**

A scoping review was conducted, and a total of 38 articles met the inclusion criteria and were analyzed using Bowser and Hill's framework of the seven typologies of Disrespect and Abuse (D&A) in childbirth. D&A in childbirth (or violations to RMC) is a manifestation of OV and served as a proxy to analyze its prevalence in the EMR.

**Findings and Discussion:**

This study indicated that across the EMR, women experienced every type of D&A in childbirth. This happens regardless of health systems' strength or country's income, with 6 out of 7 types of D&A found in almost two-thirds of included countries. In the EMR, the most common types of D&A in childbirth are physical abuse (especially overused routine interventions) and non-dignified care (embedded in patriarchal socio-cultural norms). The intersections of these abuses enable the objectification of women's bodies and overuse of unconsented routine interventions in a hierarchical and patriarchal system that regards the power and autonomy of doctors above birthing women. If unchecked, the implications include acceptance, continuation, and underreporting of D&A in childbirth, as well as passivity toward human-rights violations, which all further cause the continuing the cycle of OV.

**Conclusion:**

In order to eliminate OV, a paradigm shift is required involving infrastructure changes, education, empowerment, advocacy, a women-centered and gender-sensitive approach to health system strengthening, and policy development. Recommendations are given at individual, community, health systems, and policy levels to ensure that every woman achieves her right to health and birth in a dignified, respectful, and empowered manner.

## Background

In order to achieve the right to health and Universal Health Coverage (UHC), all people must access high-quality, appropriate, acceptable, and essential health services without suffering financial hardship or impoverishment, and this includes maternal care (1). Women around the world deserve the right to access high-quality maternal care, defined as safe, effective, efficient, equitable, timely, and patient-centered (2, 3).

**Figure 1:**
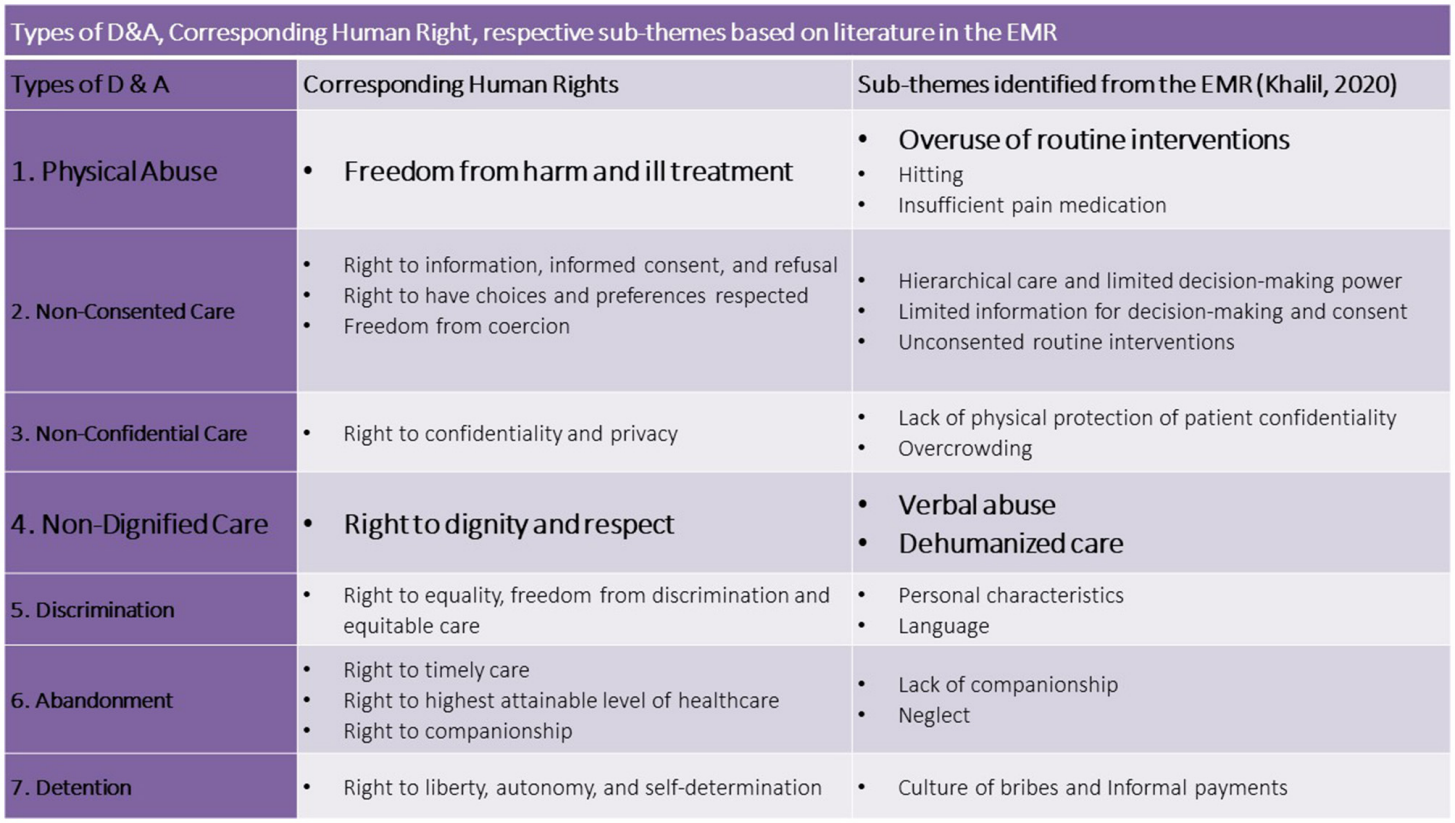
Bowser and Hill (B&H) Framework defining categories of Disrespect and Abuse in Childbirth and respective human rights violations, adapted from Bowser and Hill, 2010. Source: Respectful Maternity Care: The Universal Rights of Childbearing Women—Maternal Health Task Force.

Obstetric violence (OV) is a form of gender-based violence (GBV) that targets pregnant and childbearing women during and beyond the intrapartum period, and this case violates human rights and evidence-based medicine and hindering the delivery of respectful maternity care (RMC) (4, 5). The gendered, structural, and institutional nature of OV makes it difficult to recognize due to its widespread normalization and embeddedness in health systems and socio-cultural norms (6–8). OV is manifested through disrespectful and abusive intrapartum care, and it results in women's dissatisfaction with the poor quality of care. This circumstance ultimately influences their decisions in delaying or avoiding the use of health services in subsequent pregnancies and births, which undermines global efforts to reduce maternal mortality and achieve UHC (9–11). In inaugural landscape analysis, Bowser and Hill (B&H) identified seven categories of disrespect and abuse (D&A) in childbirth, each correlating to one or more human rights, which will serve as the analytical framework for this study (12, 13) (Figure 1). OV remains a new concept in global health literature with scholars utilizing various terminologies to define it. For example, some defend the use of the term “obstetric violence” to stress its structural nature as a form of GBV, while others use “mistreatment,” “dissatisfaction,” or “D&A in childbirth” to capture various nuances in its manifestation and subjectivity in how birthing persons experience it. The interconnectedness of these concepts is explained further below.

**Figure 2:**
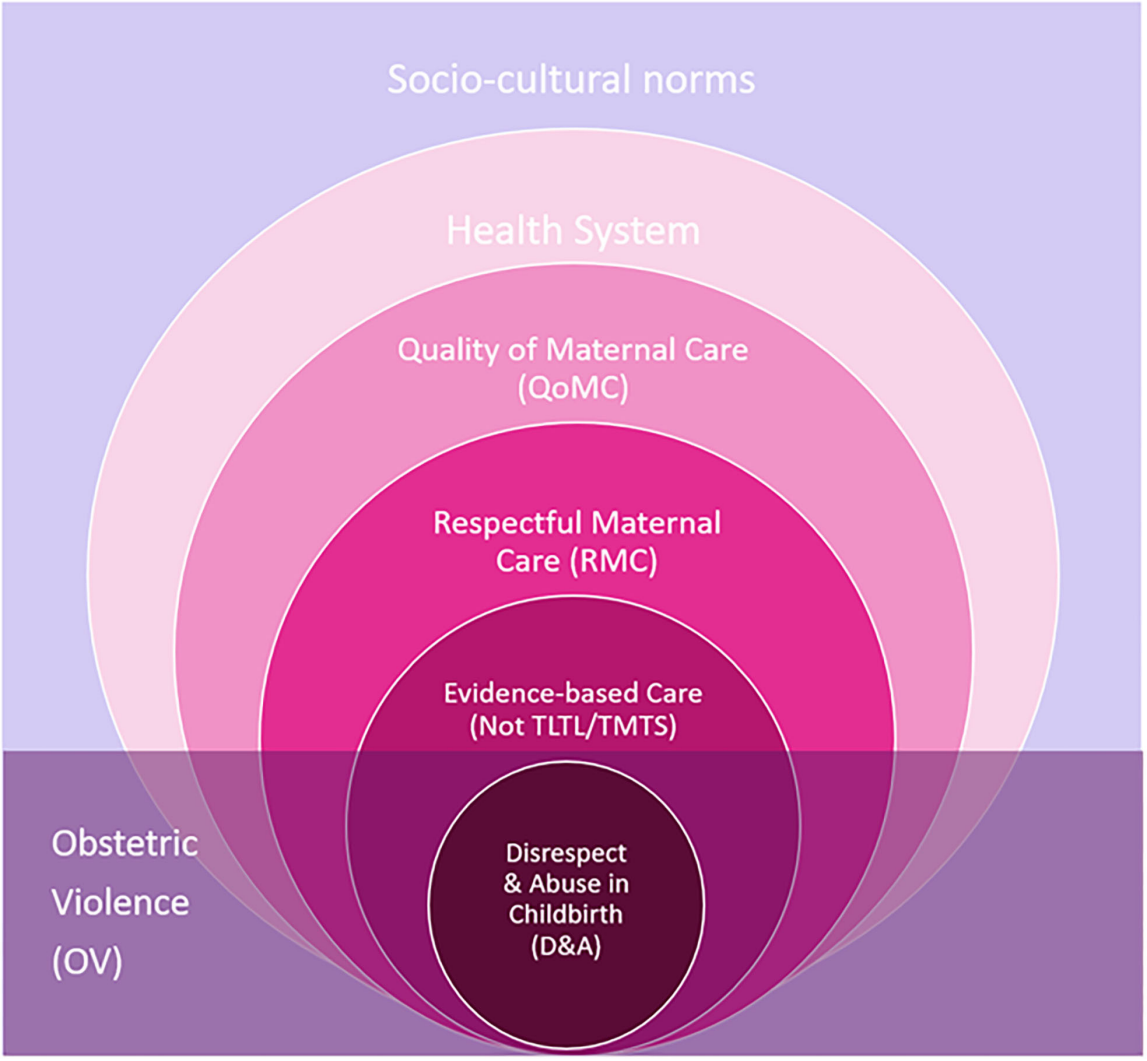
Conceptualizing Obstetric Violence and its relationships with D&A, RMC, QoMC, health systems, and socio-cultural determinants.

Globally, safe motherhood initiatives and WHO guidelines have resulted in 75% of women giving birth with a skilled-birth attendant (SBA), among which 66% having at least 4 antenatal (ANC) visits and about 50% having facility-based deliveries (FBD) (with differences based on socio-economic status) (10, 11). Despite these advances, women worldwide continue to suffer due to delays, ineffective, inefficient, inadequate, unnecessary, harmful, or disrespectful services, indicating the need to improve the quality of maternal care (QoMC) (1, 9, 10). Some health systems barriers to QoMC include the lack of women (patient)-centered care approaches, inadequate staff, deficient training, outdated equipment, limited supplies, rundown infrastructure, and insufficient evidence-based clinical guidelines or more often inadequate adherence to existing guidelines (9, 10). Additionally, poor QoMC is usually also associated with deviation from evidence-based guidelines where care is either “Too Little Too Late (TLTL)” (i.e., inequitable and untimely access to services, resources, health workers, and information, resulting in maternal deaths or near-misses and morbidity) or “Too Much Too Soon” (TMTS) (i.e., expensive, potentially harmful, overmedicalization and poor regulation of interventions often resulting in birth trauma and reduction of QoMC) (10). In efforts to improve QoMC, global maternal health scholars created the Respectful Maternal Care charter, recognizing it as a universal human right for every childbearing woman in every health system globally (4, 5). In operationalizing RMC and subsequently tackling D&A in childbirth, scholars recommended the “use of evidence-based guidelines to tackle TMTS and TLTL, coupled with efforts to ensure that respect and dignity are integral parts of QoMC that women should receive throughout pregnancy, childbirth, and the postnatal period” (10). Furthermore, the coupling of these non-evidence-based practices (TMTS/TLTL) with D&A care underpins the manifestation of OV and results in inadequate QoMC, which has implications on poor maternal and child clinical outcomes. This may contribute to generational birth trauma, and subsequently, result in mistrust and underutilization of health systems (12, 13). The conceptual framework (Figure 2) shows how OV intersects with the non-evidence-based practices, human rights (including more broadly the right to RMC), health systems barriers affecting QoMC, and gendered socio-cultural norms which cross-cuttingly influence health systems users and providers. D&A in childbirth (or violations to RMC) is a manifestation of OV, which is shown by OV completely overlapping the D&A circle in Figure 2. For the purpose of this study, D&A will be used as a proxy to explore the prevalence of OV in the Region.

The Eastern Mediterranean Region (EMR) comprises 22 countries, from Morocco to Pakistan, and is characterized by diversity in demographics, socioeconomics, security, and health systems from relatively stable to fragile (Table 1). At the time of this study, there are almost 583 million people living in the Region, among which the majority are youth (under 30), with 1/5 of the population being adolescents between 13 and 18 years of age. Total fertility rates range from 1.5 children per woman in the UAE to 6.4 in Somalia, while adolescent fertility rates range from 0.1 per 1,000 girls aged 15–19 in Lebanon to 87 per 1,000 in Afghanistan (14). With regards to reproductive and maternal health indicators, the EMR ranks second-worst and has among the highest rates of maternal and neonatal mortality globally (15). In the EMR, there is generally a culture of silence around Sexual and Reproductive Health and Rights (SRHR) which are considered taboo, confounded by a generally patriarchal and conservative culture that sets rather strict gender norms and roles and affects women's perceptions of their bodies, their autonomy over them, their health literacy on SRHR issues, and their health-seeking behaviors (16, 17). In many countries in the EMR, the concepts of informed and shared decision-making are a new phenomenon since decision-making power on matters of maternal health (and SRHR) remains often with family members who are men, doctors, and religious leaders (16–18).

**Table 1:** Countries in the EMR (14, 15).

**High Income (HIC)**	**Middle-Income (MIC) (Upper and Lower)**		**Low-Income (LIC)**
		**WHO Emergency Operations**	
Bahrain	Djibouti	Iraq	Afghanistan
Kuwait	Egypt	Libya	Somalia
Oman	Iran	Pakistan	Sudan
Qatar	Jordan	Palestine	Syria
Saudi Arabia	Lebanon		Yemen
United Arab Emirates (UAE)	Morocco		
	Tunisia		

Global evidence suggests that OV and D&A in childbirth are ubiquitous in every health system (19). WHO's recent multi-country study in 4 lower-middle income counties (LMICs) (Ghana, Guinea, Myanmar, and Nigeria) found that over a third of women experienced D&A in childbirth, with young women being 1.8 times more likely to experience physical abuse, and less educated women 3.6 times more likely to experience verbal abuse. Furthermore, unconsented care was frequently reported with 60% of participants subjected to unconsented vaginal exams, 75% episiotomies, 27% inductions, and 11% C-sections (20). Across the EMR, the provision of technocratic maternal care (often TMTS) is seen with high rates of routinely used inductions, amniotomy, c-sections, and episiotomies during childbirth (10). The majority of published evidence on OV and D&A is concentrated either in Sub-Saharan Africa (SSA), North America, or Europe, with one global systematic review on RMC included only four articles from the EMR (19).

Historically, QoMC researchers have used outcome (e.g., maternal mortality and births attended by SBA) or process/output indicators (e.g., c-section rates by wealth-quintile, length of hospital stay after birth, availability of emergency obstetric, and newborn care). However, these indicators require further development, standardization, validation, and revision as they do not address the issues of timeliness of services, costliness, provider skills/attitudes, or women's perceptions and satisfaction (1, 21). Moreover, the objectification of women's bodies as an instrument ensuring the safe delivery of their babies undermines women's feelings of satisfaction during their births and their experiences of respect in childbirth (22, 23). Additionally, data on non-evidence-based care (TLTL/TMTS) usually focuses on the one-angle, provision of health services, without sufficiently capturing women's experiences of childbirth (10).

Since 2019, UN and global players advocated and recognized the urgency of addressing OV in hopes of dignifying the experiences of childbearing women, protecting women's rights, and improving QoMC toward advancing UHC (8). Given this global momentum and dearth of evidence on OV regionally, this study aims to center the experiences of women's mistreatment in childbirth to provide an overview of OV and patterns of D&A in the EMR and offer recommendations for policy and practice on improving RMC in the region.

## Methods

This study follows a descriptive study design based on available peer-reviewed and gray literature. Guided by Arksey and O'Malley's five-stepped framework, a scoping review was conducted to answer the following research question: How is obstetric violence experienced in the EMR?

To identify relevant publications, a search of PubMed, CINAHL/EBSCO, Google, Google Scholar, VU Libraries, Cochrane Databases, Harvard Maternal Health Task Force (MHTF) Publications, and the Eastern Mediterranean Health Journal (EMHJ) was conducted. The following PubMed search strategy was used: “((((“disrespect and abuse”[All Fields] OR (obstetric[All Fields] AND (“violence”[MeSH Terms] OR “violence”[All Fields]))) OR (respectful[All Fields] AND (“mothers”[MeSH Terms] OR “mothers”[All Fields] OR “maternal”[All Fields]) AND care[All Fields])) OR (“parturition”[MeSH Terms] OR “delivery, obstetric”[MeSH Terms])) OR (mistreatment[All Fields] AND (“pregnant women”[MeSH Terms] OR (“pregnant”[All Fields] AND “women”[All Fields]) OR “pregnant women”[All Fields]))) OR “patient satisfaction”[MeSH Terms] AND (“middle east”[MeSH Terms] OR “africa, northern”[MeSH Terms] NOT “Turkey”[All Fields]) NOT “Israel”[All Fields] AND “2010/04/23”[PubDate] : “2020/04/19”[PubDate].” To identify other peer-reviewed and gray literature, the following keywords and their variations on “obstetric violence,” “Disrespect and Abuse in Childbirth,” “Respectful Maternal Care,” and “Dissatisfaction in intrapartum” in each of the 22 countries in EMR were used to search the other databases and search engines (Supplementary Table 1). Systematic reviews and peer-reviewed publications were snowballed, and reference lists were also screened to identify frameworks used in the global literature, articles relevant to the EMR, and experiences of other LMICs for comparison.

**Figure 3:**
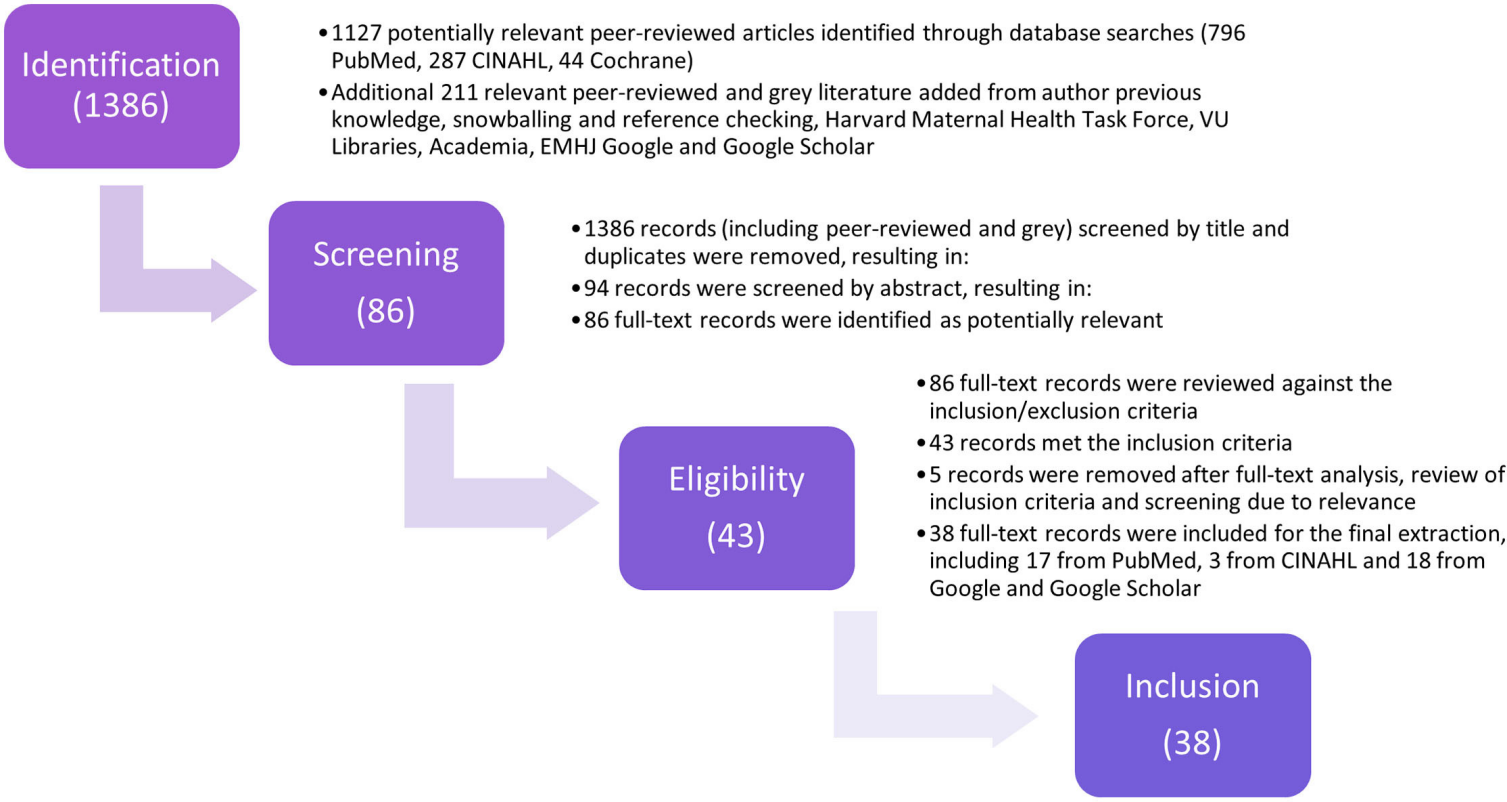
PRISMA flowchart of search and screening strategy.

Regarding the screening and selection of studies, literature was searched between April and May 2020. The inclusion criteria comprised of articles published in English. Although the EMR's main languages also include Arabic and French, an initial search of literature published on this topic indicated that publications (peer-reviewed and gray) in these other languages were few in quantity, of weak methodological rigor, usually direct translations of English counterparts, or not relevant to this study. Only articles published starting 2010 were reviewed as this was when the B&H landscape analysis was published, and a cap of 10-year was prioritized to remain within the more updated publications. Regarding the geographic context, while various geopolitical arrangements exist to define the “Middle East and North Africa Region,” WHO's “regional division” was used for this review, limiting the search to the 22 EMR member states (excluding Algeria, Israel, and Turkey). Articles related to the EMR diaspora were also excluded. In applying a feminist approach and in hopes of capturing and highlighting the voices and perspectives of women in the EMR related to their childbirth experiences, articles were excluded if related to provider knowledge, attitudes, skills or malpractice in intrapartum care, randomized clinical trials, postpartum outcomes, quantitative QoMC indicators (e.g., ANC, FBD, and SBA), or assessment tools. Following title, abstract, and full-text screenings, a total of 38 records were extracted (Figure 3).

Following this study's analytical framework (Figure 1), the data were extracted according to B&H's seven categories of D&A (Supplementary Table 3). In hopes of positioning and comparing the situation of D&A in the EMR within the global landscape, this landmark framework was selected due to its simplicity and its frequent use in the global literature. Nevertheless, as OV is manifested at the intersection of socio-cultural norms and the health systems, an additional column was added to the extraction table titled “other” for findings that did not correspond explicitly to the seven categories.

The findings were summarized according to the seven types of D&A and other additional drivers. Following this initial charting of findings, sub-themes were deductively synthesized from the literature and cross-checked with various systematic reviews to address the overlap of sub-themes between typologies (10, 19, 21, 28).

## Findings

The search initially yielded 1,386 articles, of which 38 records were included for analysis (Figure 3, Supplementary Table 2). Among these 38 articles, 35 (90%) were peer-reviewed publications, 14 used qualitative methodologies, 14 used quantitative methodologies, five used mixed-methods methodologies, and two were literature reviews (Supplementary Table 2). Three non-peer-reviewed records were added, namely an NGO report, a Ph.D. thesis, and a commentary by a birth doula based in the EMR, due to their explicit relevance to this study scope.

Among the 38 articles meeting the inclusion criteria, 60% (23/38) of publications were published between 2017 and 2020. Three articles provided a multi-country or regional analysis, while 35 were country-specific. Two-thirds of the region was represented in the literature with half of the Region (11/22) having country-specific publications. A quarter of countries in the EMR (6/22) had 4–5 country-specific publications included in this analysis, indicating that despite limited literature, there is not one specific country bias in regional literature (Table 2). Egypt, Iran, Jordan, and Saudi Arabia had the highest and an equal number of publications, likely since these countries contribute to a bulk of evidence-generation and publications in the EMR (24). Most articles were based on large, public, urban hospitals (Supplementary Table 2). Almost all included publications were written by teams of authors from the Region.

**Table 2:** Number of articles mentioning the categories of D&A in the EMR literature.

**By Country**	**Income Standing (World Bank classification)**	**1. Physical abuse**	**2. Non-Consented care**	**3. Non-Confidential care**	**4. Non-Dignified care**	**5. Discrimination**	**6. Abandonment**	**7. Detention**	**Total Types mentioned (x/7)**	**Total Number of included articles**
Afghanistan	LIC	2	-	1	3	-	3	2	5	4
Egypt	MIC	4	3	2	5	1	4	-	6	5
Iran	MIC	3	2	1	2	1	1	-	6	5
Iraq	MIC	2	2	3	4		2	1	6	4
Jordan	MIC	3	3	4	3	1	4		6	5
Lebanon	MIC	1	1	-	-	-	-	-	2	1
Pakistan	LIC	2	2	2	1	1	1	1	7	2
Saudi Arabia	HIC	4	3	4	4	1	4	1	7	5
Sudan	LIC	2	2	2	2	1	1	1	7	2
Tunisia	MIC	1	1	-	1	1	-	-	4	1
Yemen	LIC	1	1	-	1	-	1	-	4	1
Total number of countries^*^ mentioning Type of D&A		11	10	8	10	7	9	5	7/7	N/A
Total number of country-specific studies with Type of D&A		25	20	19	26	7	21	6	7/7	35
MC added^**^		2	3	2	2	-	2	-	5	3
Total number of studies with Type of D&A^**^		27	23	21	28	7	23	6	7/7	38

*LIC, Low-income country; MIC,Middle-income country (includes upper and lower middle income standing); HIC, High-income country*.

* *not counting multi-country studies*.

** *Multi-country studies from the Region (MC)*.

*Egypt, Lebanon, Palestine, and Syria (25). Egypt, Lebanon, Syria (26). Egypt, Jordan, Lebanon, Qatar, Saudi Arabia, and United Arab Emirates (27)*.

Literature shows that women from the EMR have experienced all seven types of D&A. Most country-specific studies mentioned at least 4 of the 7 types of abuses with the majority mentioning 6 of the 7 (Table 2). Physical abuse and non-dignified care were most frequently mentioned in the literature. Detention was the least mentioned, which is comparable to findings from numerous studies in the African Region (20, 28). Literature from Pakistan, Saudi Arabia, and Sudan mention all seven categories of D&A because studies from these countries used the B&H model as their analytical framework. Most studies are set in public urban tertiary hospitals, which confirms the deviation from evidence-based medicine in many hospital-based deliveries across countries of all incomes in the Region.

This review identified various sub-themes under each category of the seven typologies of D&A (Table 3). Notably, as with most forms of GBV, these abuses usually do not occur in isolation but often manifest in additive and intersectional manners which often makes them difficult to segregate and measure (18, 23, 29).

**Table 3:** Types of D&A and respective sub-themes based on literature in the EMR.

**Types of D&A**	**Sub-themes identified through this literature review**
1 Physical Abuse	• Overuse of routine interventions • Hitting • Insufficient pain medication
2 Non-Consented Care	• Unconsented routine interventions • Hierarchical care and limited decision-making power • Limited information for decision-making and consent
3 Non-Confidential Care	• Lack of physical protection of patient confidentiality • Overcrowding
4 Non-Dignified Care	• Verbal abuse • Dehumanized care
5 Discrimination	• Personal characteristics • Language
6 Abandonment	• Lack of companionship • Neglect
7 Detention	• Culture of bribes and Informal payments

### Physical Abuse

Physical abuse was the second most referenced form of D&A in the regional literature, mentioned in 27 studies in 15 countries (Table 2). While only a few studies in the EMR use the B&H model to measure D&A, they all indicated an overall prevalence of 6.5% (*n* = 459) of Iraqi, 8.3% (*n* = 263) of Sudanese, and 18.6% (*n* = 360) of Pakistani women who experienced physical abuse in childbirth (30–32). This review identified the following sub-themes: overuse of routine interventions, hitting, and insufficient pain relief.

While the overuse of routine interventions, or more broadly technocratic childbirth models, can be a cross-cutting sub-theme as it violates almost all corresponding human rights (Figure 2), the authors opted to include this sub-theme as a form of physical abuse in childbirth highlighting the physiological and biomedical intrusions underpinning subsequent psychological trauma which may compound with other forms of D&A. In the EMR, there is a tendency for obstetric care to be technocratic, overmedicalized with non-evidence-based interventions routinely pushed on women (TMTS) (25, 27, 33–37). While routine obstetric interventions have historically been associated with westernized medical models and higher-income health systems; in the EMR, this review revealed their overuse across low (Afghanistan, Pakistan, and Sudan), middle (Egypt, Jordan, Lebanon, Palestine, and Syria), and high (Saudi Arabia, Qatar, and UAE)-income countries (6, 10, 25, 27, 30, 38) (Supplementary Table 3).

Across the region, episiotomies and inductions are routinely utilized in parturition (25, 27). One woman from a MIC mentioned: “*As soon as I got to the hospital, he ordered the induction straight away”* (36), indicating over-medicalizing and over-managing second-stage of labor. In some of the Region's MICs [e.g. Egypt (33), Jordan (39), and Iran (35, 40)], these unnecessary inductions have resulted in readmission due to contracted infections. Another study from Afghanistan documents women compared doctors to butchers, emphasizing this forceful and aggressive behavior in cutting episiotomies and c-sections (41). Bed confinement, with limited mobility, is also routinely practiced, having bladder catheters inserted rather than allowing women to walk to the bathroom was subsequently mentioned by women in Egypt (33, 34), Iran (35), Jordan (36), Saudi Arabia (37, 42) and Yemen (43). Moreover, most of the women birthing in hospitals across the Region are not granted the choice of preferred positions in labor and birth due to hospital policies and routine practices favoring the routine use of lithotomy in childbirth (44). Women are forced to give birth on their backs which often delayed labor and results in the use of other interventions; leaving women often complaining of dissatisfaction, restriction, and a sense of powerlessness and exposure (32, 43). One regional systematic review mentioned women feeling violated as health providers forced their legs open for vaginal exams and births (27). Furthermore, the normalization of frequent vaginal exams was reported by women to increase their fear as they consider them more painful and traumatic compared to labor and birth (27, 33, 37). A birthing woman in Jordan recounted: “*I did not receive any care other than having vaginal examinations frequently”* (36) while women in Pakistan reported requesting C-Sections to avoid being violated by health workers and traumatized by frequent vaginal checks (45).

Secondly, physical abuse in form of hitting was commonly reported in EMR countries of varying income-levels (Supplementary Table 3). For example, in studies from Afghanistan, patients reported incidences of hitting, slapping, or being pulled by the hair by doctors and midwives (38, 46). An interviewee from Tunisia reported “*being beaten on the hips, slapped on the face and having finger marks on her body*.”(47).

Thirdly, women in the EMR have expressed not receiving sufficient pain medications and indicated feeling neglected by health workers when in pain, which intersects with D&A Type 6 Abandonment. One Saudi woman reported “*they started cutting the incision and I felt the scalpel and the stretching; of course, I screamed very loudly. Finally, they said fine and gave me complete anesthesia*” (48). Another study from Iran indicated women being induced artificially without the provision of adequate pain relief (40). Intrapartum survey-respondents in Iraq, Jordan, and Saudi Arabia were dissatisfied by the availability and strength of pain medications (Supplementary Table 3).

### Non-consented Care

Non-consented care was mentioned in 23 studies and at least 10 countries in the EMR (Table 2). The subthemes for non-consented care included unconsented routine interventions (intersecting with the previous typology), hierarchical care and limited decision-making power, and limited information for decision-making and consent.

Studies from the Region using the B&H model to capture D&A, found overall prevalence of non-consented care experienced by 100% (*n* = 11) of respondents in Tunisia, 97.5% (*n* = 360) in Pakistan, and 35% (*n* = 263) in Sudan (30, 31, 47). These small sample sizes are not indicative of prevalence across these densely populated countries but rather display cases of non-evidence-based and disrespectful care in childbirth across the Region regardless of country income standing or health systems fragility.

The normalization of overmedicalized care in the Region enables women to expect overused routine interventions. However, the problem extends that these interventions are commonly done without women realizing or consenting (27, 33, 35, 42) (Supplementary Table 3). For instance, one study from the largest medical complex in Saudi Arabia found 19% (*n* = 358) of women underwent episiotomies, 3.6% underwent c-sections, 1.4% underwent tubal ligations, and .3% underwent hysterectomies, all of which had routinely administered without consent (49). In Iran, although routine inductions and episiotomies are expected, women reported not being informed of being cut, which was disrespectful and dehumanizing (40). Another study from a large urban teaching hospital in Sudan identified a notable discrepancy regarding consent and type of intervention as 77.5% of study-participants were asked permission for an exam but only 22% before a procedure (50). Another study reported a midwife inserted a urinary catheter without the woman's consent, causing violation and pain, in an effort to prevent her from pushing before allowing the woman to use the toilet; the same study highlighted providers' rationalizing these unconsented routines with the hospitals' policies to maintain patient flow (42).

The seldom-questioned hierarchy and power relations in health care settings between physicians, hospital managers, nurses, and midwives, place birthing women at the bottom of the chain (7, 27). Lowest-ranked in the medical hierarchy, and vulnerable due to patriarchal and gendered dynamics of obstetric care, birthing women in the Region often feel inferior to doctors, discouraged to participate in decision-making processes, intimidated to ask questions fearing to be shamed or insulted, and passively under their authority (18, 44, 46, 51). One Irani woman confirmed: “*Women are not involved in decision-making, they trust their caregivers to make decisions for them and don't challenge their [providers] who are more knowledgeable*” (40)., One Yemeni woman highlighted that “*to be in-authority meant sharing decision-power with the care provider*” (26, 43). Shared decision-making remains a novel concept to the Region's generally patriarchal health systems, as physicians do not expect women “to interfere” with their medical practice and often follow their own decisions rather than hers (42). The lack of decision-making power and bodily autonomy especially at birth leaves women feeling powerless as reported extensively across the Region (Supplementary Table 3).

Many women in EMR reported feeling dissatisfied with the information received from their health providers, often disappointed by the types and amounts of information presented to them, leaving them disempowered to make any decisions and feeling objectified as procedures were being done to their bodies (Supplementary Table 3). A study on verbal and non-verbal abuses in public urban facilities in Iraq found that almost half of participants were dissatisfied with the provider's assessments, explanations of diagnosis, and untailored treatments (52). Women in Saudi Arabia expressed their providers did not provide information before injections or stitches nor explain aftercare (48). Others were not offered options or alternatives in childbirth, such as in Lebanon where women felt they were robbed of the opportunity to make informed-choices about their c-sections (26, 53). In some cases, studies mentioned doctors use laboring women's vulnerabilities to coerce them or make the decisions on their behalf often without asking consent or providing adequate information for the patient to make informed decisions (27, 33). This hierarchy in patient-provider relationships and the lack of communication caused women to feel dominated and disrespected as passive subjects receiving care; this non-consented care enables various types of D&A to occur across EMR countries of all contexts (33, 43, 46, 49).

### Non-confidential Care

Women perceived privacy (whether physical or informational) and autonomy in birth among the most important elements of QoMC. However, non-confidential care was reported by women regardless of the types of facility and country-income, in 21 studies in at least 8 countries (Table 2, Supplementary Table 3) (40, 45, 54, 55). This review identified lack of physical protection of patient confidentiality and overcrowding as sub-themes for non-confidential care.

In Afghanistan, Egypt, Pakistan, and Sudan, shortages in beds, curtains, and equipment had resulted in women birthing on the floor, in corridors, or sharing birthing spaces which contributed to discomfort and privacy violation (27, 31, 45, 54). A national study from Afghanistan found that visual and auditory privacy in facilities was available in half of the facilities for antenatal consultation rooms, and only 60% in the delivery room (54). On the other hand, women in Saudi Arabia, also faced this problem as 5.6% (*n* = 358) of their population gave birth in rooms without curtains separating beds (49). In the case where appropriate furniture was available, differences are observed in confidential care between urban-rural and hospital types. In Afghanistan, urban women felt shame to access maternity clinics in Kabul, compared to their rural counterparts, fearing loss of modesty by being exposed for exams or birthing in overcrowded and underequipped facilities (54). In Sudan, health workers were less likely to use curtains and visual barriers in a Khartoum tertiary teaching hospital compared to smaller, secondary maternity hospitals (53% non-compliant in protecting privacy in childbirth compared to 6.8% in the latter) (31). Breeches in patient confidentiality were further reported whether in discussing private information in public, misusing filing systems, seeing patients in groups, or utilizing open-door policies in maternal hospitals (31, 37, 49, 52). Overcrowding was another commonly mentioned factor contributing to poor perception of QoMC in public hospitals in LMICs both in and beyond the EMR (1, 11, 26). Women reported sharing their labor room with other birthing women and receiving frequent unwanted visits from an overload of unknown staff (27, 34, 56). Across all income-contexts (i.e., Sudan, Jordan, and Saudi Arabia), women's privacy was interfered with by a large number of students (31, 48, 57).

### Non-dignified Care

Non-dignified care was reported as the most prevalent form of obstetric violence in the EMR (Table 2), mentioned in 28 studies and at least 13 countries. Azhar et al. found that women were 6.5 times more likely to experience abandonment in care than report it (30). The sub-themes identified were verbal abuse and dehumanized care. Notably, as with many other forms of GBV, OV can be subtle and nuanced by sociocultural definitions and attitudes which further make it difficult for many women to recognize, name, or disclose.

Across various types of facilities from major tertiary hospitals to health center and regardless of income, women in all country-contexts reported experiencing elements of dehumanized birth (Supplementary Table 3). These various elements were reflected as receiving obstetric care from unfamiliar providers, who often did not introduce themselves, used demeaning verbal and non-verbal cues further creating an unwelcoming environment and distance in the patient-provider hierarchy. Women also described feeling neglected by staff at health facilities, having to beg and plead for timely attention from providers, not receiving adequate pain management, or having to give birth alone due to delays in receiving care. Women further reported treatment by staff as “unfriendly,” “unprofessional,” “rude,” “unempathetic,” “hostile,” “absent,” “impersonal,” and “authoritative” (27, 43, 46, 52). Subsequently, undignified care compromises QoMC, access, compliance, and effectiveness as women fear humiliation, neglect, loss of control, and disrespect (21, 33, 56).

Regardless of parity and stage of labor across EMR countries, verbal abuse was the most common form of undignified care reported by women. Examples in Supplementary Table 3 show a wide range of experiences; variations also depend on the definition/inclusivity of “verbal abuse,” methodology, and sample size of studies. Being scolded, belittled, insulted, shouted at, gaslighted, and threatened while laboring in pain were examples given across EMR countries, regardless of income-standing (27, 46, 47, 58). Studies across high and LMICs mentioned experiences of women being gaslit, called liars, and threatened to “*stop whining like children or leave the hospital*” (48) or “*I will cut your vagina [episiotomy], if you don't push good*” (59). This abuse was exacerbated for women whose intersectional identities left them more vulnerable, for instance, pregnant Afghani refugees in Iran reported verbal discrimination: “*You behave like a donkey*” (46), “*if you can learn to get pregnant, you should learn to tolerate pain*” (54). Further examples of discrimination, and the overlap between these typologies are explained below.

### Discrimination and Detention

Discrimination and detention were among the least frequently reported themes across the regional literature (Table 2). Furthermore, one study highlighted the discrepancies in prevalence data, as women were five times less likely to report discrimination compared to experiencing it (30). Subthemes identified included discrimination based on personal characteristics and language and detention due to financial debts (Table 3).

Studies using the B&H model to measure D&A found that detention due to inability to pay was reported by 1.9% (*n* = 263) of women from Sudan, 1.4% (*n* = 358) in Saudi Arabia, and 0%(*n* = 360) in Pakistan (30, 31, 49). On the other hand, expected informal payments to ensure timely attention from care providers and access to medications contribute to women's feelings of extortion, and are reported among the highest causes of dissatisfaction in maternal care (41). Bribes are common-practice in the Region, not only in obstetrics or medicine as reported by parturient women in countries of all income-groups (Supplementary Table 3) (46). In Sudan, a third of women who reported discrimination attributed it to their income, while in Pakistan, women with lower socioeconomic status were three times more likely to experience D&A (30, 31).

Moreover, beyond financial discrimination which predominantly affects the poor, global literature expands the definition to encompass differences in treatment based on personal characteristics, including age, income, race, or marital status, among others (20, 21, 28). Experiences from the Region are consistent with global trends, as younger women (often first-time mothers) in Jordan were more likely to experience verbal abuse (20, 59). Due to the conservative culture of the Region and the high stigma against pregnancy outside of wedlock, single mothers in Tunisia experienced unfair treatment or worse, were denied treatment (47). On the other hand, refugees and non-nationals often experienced covert discrimination and disrespect based on their nationality, including denial of admission, delayed care, or mistreatment by staff (14).

Additionally, despite the right to access health services in a language that they understand, women across the Region mentioned the use of inaccessible medical jargon and use of the non-native tongue (13) (Supplementary Table 3). One study from Saudi Arabia found that medical staff intentionally spoke in English to exclude patients from decision-making (37).

### Abandonment

Literature from the Region mentions abandonment as a form of D&A in 23 studies in at least 14 countries, with sub-themes of lack of companionship and inadequate attention from medical staff (Tables 2, 3). In the Region's largest country, Pakistan, women were 6.5 times more likely to experience abandonment in care than report it (30).

In many EMR countries, hospital policies generally prevent women from being accompanied by partners, doulas, and labor and birth companions leaving women to labor and give birth alone, thus contributing to increased feelings of isolation, pain, and abandonment (26, 32, 43, 60, 61). This restrictive hospital policy was found to be prominent in the regional literature in Afghanistan (38), Iran (61), Iraq (32), Jordan (36, 56), Lebanon, Syria, Egypt (26), and Saudi Arabia (37, 48), further examples are documented in Supplementary Table 3. Where exceptions to these policies allowed women to have a family member or support person present, health care providers in low (38), middle (56), and high (37) income countries discouraged or denied their entry fearing they will inhibit sterility, cause conflict, and reduce QoMC (27).

Studies displayed abandonment by staff across all intrapartum stages, expressed in three forms: insufficient, untimely, and unempathetic care. Women, in all income contexts, had to beg health workers to attend to their care, contributing to women feeling not prioritized and disrespected. One Saudi woman recalled: “*I was in pain and I almost kissed their hands to check me. I kept bothering them until they examined me and they found that I was 8 cm dilated”* (48). Untimely care was expressed by women, across the Region regardless of country-income, and at an extreme, resulted in women giving birth alone without a medical provider present (Supplementary Table 3). A mother in Lebanon recounted: “*when I was in labor, the nurses used to leave me and watch TV.”* (27). From Jordan, a new mother reported: “*I was left alone in the labor room, I felt my baby coming out*” (56), while in Afghanistan, “*I delivered on the floor of the corridor while another patient called out for the doctor or cleaner*” (41). These disruptions in continuity of care and ignored requests for support or pain management contributed to women's reported sense of abandonment and poor QoMC.

Notably, studies from both Egypt and Jordan provide conflicting inter and intra-country findings of women's perceptions of empathy and kindness from providers. Abdel Ghani and Berggren, Monazea and Al-Attar, and Elgazzar et al. reported that two-thirds of participants in Egypt prioritized the need for nurses to demonstrate empathy in care while two other studies support this as 39.1% (*n* = 435) and 37.4% (*n* = 214) were dissatisfied due to poor emotional support from nurses (34, 62, 63). Another study in Egypt (*n* = 501) found that about 75% of respondents were treated kindly/friendly by health workers, but about half felt that staff did not treat them empathetically or respectfully (64). Similarly in Jordan, Hatamleh et al. found that 64% (*n* = 460) of women reported friendly/polite treatment but 31% felt disrespected, and 36% were verbally abused or neglected (36). These differences in experiences further confirm the complex nuances which may discredit women's feelings of disrespect and abuse at birth.

### Other Multi-Level Drivers of D&A

In addition to the sub-themes for each of the 7 D&A typologies, this review also uncovered multi-level drivers enabling the normalization of OV in the EMR including personal, health systems, and socio-cultural factors, in line with new frameworks exploring mistreatment in childbirth (7, 21, 65–68).

Firstly, Ghanbari-Homayi et al. and Kempe identified other significantly associated personal factors for traumatic birth experiences including the following: marital dissatisfaction, lack of insurance, poverty, unwanted pregnancy, fear of childbirth, spirituality/faith, individual perceptions of pain, and companionship (35, 43). Further, given that two-thirds of the Region is affected by conflict, the intersecting identities of displaced and refugee women (particularly young, poor, uneducated, unaccompanied, and survivors of war) leave them particularly vulnerable to all forms of GBV including OV (14, 20, 47).

Secondly, health system factors are complex and affect various aspects of QoMC; nonetheless, the overall lack of gender-sensitive approaches to health systems management in the EMR underpin the normalization of OV (18, 67). In the EMR, poor infrastructure, limited bed capacity, lack of supplies and medicines, and unhygienic facilities were mentioned as factors for dissatisfaction and causes for the low-utilization of public facilities in Afghanistan, Egypt, Iraq, Jordan, and Pakistan (30, 34, 39, 54, 69). Systematic reviews in LMICs, especially from SSA, confirmed these barriers to RMC (3, 7, 21, 28). Childbirth in public facilities and/or teaching hospitals was also associated with higher levels of D&A as in Iran, Jordan, and Pakistan; while in Lebanon, the health system is predominantly run by private-sector, which confounded the causes for overmedicalized care and overuse of routine interventions, like C-Sections (32, 35, 39, 53). In countries fragile and conflict-affected settings such as Afghanistan, Iraq, and Yemen, insecurity, displacement of persons, high workload and shortages of health providers, lack of infrastructure, and limited supplies affect quality and women's experience of care (46, 70, 71). In addition to overall shortages in maternal health workers, especially nurses and midwives in many EMR countries, the lack of access to female physicians was cited as a barrier to providing culturally-acceptable care in a generally culturally-conservative Region (34, 54). Many women have expressed greater satisfaction working with (predominantly female) nurses and midwives as compared to physicians, as in Egypt and Saudi Arabia; while in Jordan, a significant association was found between the experience of abuse and type of health provider (48, 59, 62). Women across the Region empathized with health workers due to harsh, hierarchical, and often abusive working conditions (7, 34, 41, 46, 54, 72). Moreso, studies from both Egypt and Jordan indicated the need for refresher pieces of training for nurses, not only on basic clinical skills to avoid reliance on traditional practices or experience but also on psychosocial/emotional care (34, 56). On the other hand, insufficient health workforce attitudes and competencies in providing women-centered care likely exacerbate OV. For example, Shaban et al. concluded that many doctors do not have the skills to manage birth without cutting episiotomies (39). In Egypt which ranks among the highest rates of C-Sections both globally and regionally, the overuse of this intervention was attributed to poor training and supervision, financial incentives and convenience, and limited awareness of clinical guidelines (75).

Thirdly, socio-cultural factors are among the most significant enablers to OV (Figure 2). Obstetric violence is embedded in the socio-cultural determinants which may normalize the mistreatment of women, set the norm for gender roles in the home, society, and health system, and dictate society's perceptions of control, violence, power, rights, and subsequent hierarchies (74, 76, 77). Moreover, OV may occur while providing legally approved and internationally-recommended medical protocols; as the cultural nuances define what constitutes “respectful” and “women-centered” care (7, 26, 68, 76). Regional findings further reflect a gender gap requiring a disruption to hierarchical and patriarchal norms which limit women's autonomy over their bodies, SRHR, and choices (26, 33, 46, 51). Addressing OV requires interventions to tackle deep-rooted socio-cultural beliefs and practices at the intersection of institutional violence against women and failures of the health system to provide respectful, evidence-based, women-centered, and quality (65, 67, 68, 78).

## Discussion

This review aimed to overview the burden of OV in the EMR from the experiences of birthing women to identify gaps in RMC and QoMC. The findings of this study indicate birthing women in the EMR experienced every type of D&A, regardless of health systems strength or country-income, with 6 out of 7 types of D&A found in almost two-thirds of included countries. This review found that in the EMR, the most common types of D&A in childbirth are physical abuse (especially overused routine interventions) and non-dignified care. The power dissonance between providers and women, grounded in patriarchal socio-cultural norms, often makes it difficult for parturients to recognized nuanced OV, make informed decisions, advocate for their birth preferences, and experience childbirth non-traumatically.

Notably, the narratives and experiences of women in the EMR are personal and unique and should not be generalized to indicate the overall country-wide prevalence of D&A in childbirth. The wide differences in prevalence could be attributed to sampling sizes, provider skills and attitudes, and hospital policies between countries; nonetheless, the overuse of these interventions defies evidence-based practice and indicate OV in the EMR. Only three studies from the Region used the B&H model to analyze the magnitude of D&A; which makes it is difficult to accurately estimate the overall prevalence of OV in the Region and compare it among countries and regions. Even in global systematic reviews, the wide range in prevalence is misleading to fully capture the exact prevalence and magnitude of these violations accurately. Sando et al. reported a range of 15–98% D&A experienced in 5 countries in SSA while WHO's multi-country study found a range in prevalence from 12.2 to 98% (20, 28). Moreso, most included studies in this review are based on urban, public, (tertiary/teaching) hospitals, where a higher prevalence of D&A is observed; however, it is difficult to compare the prevalence of D&A based on facility-type and geographic distribution without further studies.

### Interpretation and Comparison With Global Experiences

Despite the diversities among EMR countries, overall, the findings of this study are comparable with the global literature. In the EMR, non-dignified care was the most common type of D&A. One possible explanation could be the broad definition, embedded in cultural nuance, ranging from verbal abuse to impersonal, unempathetic, rude attitudes, non-verbal expressions by providers, and restricted choices leading to receive care from male providers, and feel dominated, dehumanized, and objectified as a laboring and birthing woman (6, 19, 21, 26, 28). Similarly, women from Ethiopia and India ranked this type of D&A highest, possibly due to geographical and cultural proximity to the EMR (79, 80). Global systematic reviews found a high prevalence of verbal abuse across the high, middle, and low-income countries, with threats and judgmental attitudes being more common in LMICs, while objectification of women's bodies was reported more in M/HICs (19, 21, 81, 82).

Second-ranked by EMR women was physical abuse. The overuse of routine interventions was included as a sub-theme to physical abuse in this study due to its immediate violation of the right to freedom from harm (an essential principle in biomedical ethics) (83), and subsequently, other human rights violations as it intersects and compounds other types of D&A, including but not limited to the following: non-consented, non-confidential, non-dignified, and abandoned care (Figure 2). Notably, some global studies on D&A do not include it but focus only on hitting and insufficient pain-relief (Table 3) (10, 19, 21, 28). The excessive use of unnecessary interventions is the basis for the technocratic model of childbirth, which has been normalized across numerous health systems; further to this, some scholars describe this widespread phenomenon as cultural often symbolizing higher status, wealth, or higher QoMC (1, 9, 10). Literature from the EMR overwhelmingly denoted that obstetric care in the region is technocratic and overmedicalized with non-evidence-based interventions pushed on women TMTS without consent (27, 33–37). Similar trends are seen in neighboring Turkey, which shares the Region's overarching patriarchal conservative culture and trend in overmedicalization (TMTS) in birth, as routine interventions were overused (e.g., 71% inductions, 73% frequent vaginal exams, 80% restricted food/water, 75% intravenous fluids, and 70% episiotomies), resulting in significantly lower satisfaction in childbirth (83). The use of non-evidenced-based routine interventions, specifically inductions, C-sections, and episiotomies were also found in many other middle/high-income countries (M/HIC), particularly in Latin America (10, 78, 84).

Unfortunately, hospital policies denying birth companions or allowing frequent medical students rotations contribute to abandonment, inadequate and non-confidential care in many countries in the EMR. Similar experiences of neglect and delays are observed in Mozambique, Ghana, Bolivia, and 20 other countries, while refusal to provide pain relief was reported across all-income and geographic contexts (21, 85). Regarding detention and discrimination, study findings were almost identical to the global literature, especially from African countries, likely due to the narrow definition of these typologies and the widespread cultures of bribery and seniority (social-hierarchy) in many LMICs (7, 21, 28, 86).

Moreover, non-dignified care intersects almost all other types of D&A due to its roots in socio-cultural norms and perceptions of gender and power. Global scholars confirmed the need to reframe overmedicalization and OV in the context of gender equity and human rights as these structural inequalities exacerbated in many patriarchal societies enable the dehumanization and objectification of women in labor (7, 65, 78, 84). At the individual level, woman's personal identities, education, empowerment, and socio-cultural norms affect their perceptions of abuse and objectification, and definitions of “consent,” “dignity,” and “respect.” These personal attributes, combined with the normalization of OV in many health systems in the Region, make it difficult for many women to recognize its elements as D&A, define this dehumanization as OV, and report this accordingly. Further to this, it is important to note that OV does not occur in a vacuum but rather within the broader context of violence against women which plagues between 1 and 3 women globally and regionally (reported 1 in 2 during COVID-19), and this may further contribute to women's normalization of violence during childbirth (5, 18, 22, 23, 89, 90). This may explain inconsistencies in findings between experiencing disrespectful care and OV and reporting it or expressing high levels of satisfaction despite encountering elements of abuse. For example, in Pakistan, 99.7% of women experienced D&A while only 27.2% reported so, similarly in Sudan 77.2% experienced at least one type of D&A but reported 39.3, 32.3, and 5.6% low, medium, and high levels of D&A respectively (30, 31). Systematic reviews confirmed this issue in many patriarchal LMICs where women's positions are still inferior to men (19, 21). Moreso, at the health systems and national levels, the lack of accountability for medical abuses in many EMR has underpinned this form of violence against women by discrediting the victim's feelings, expectations, or perceptions of dignity/RMC compared to physicians. Due to the normalization of the culture of technocratic and overmedicalized care, it is likely that health workers are not even aware that they are perpetrating OV. This was confirmed in global literature citing that the chief barriers to improving RMC included provider attitudes, misconceptions of its definition and violations, and false beliefs that expectations are met (65, 67, 73, 74, 82). Zooming out more broadly, non-dignified care, and its intersections with other abuses is embedded in socio-cultural norms. These patriarchal and harmful socio-cultural norms are consistent with global literature which highlight these institutional and gendered structures as major drivers for D&A around the world (7, 65).

Furthermore, the structural and gendered nature of OV and its embeddedness at the intersection of the health system and socio-cultural norms normalizes its manifestations as “standards” of intrapartum care. The intersections and overlap of these typologies of abuses in the Region underpin the objectification of women's bodies and overuse of unconsented routine interventions in a patriarchal system that regards the power and autonomy of physicians above parturient women. If left unmitigated, the implications comprise (1) continuance of these abusive, overused, and unconsented routine practices by care providers, (2) acceptance, under-reporting, and lack of recognition by women and their communities, and (3) passivity toward human-rights violations by policymakers, further continuing the cycle of D&A in childbirth in the EMR.

### Strengths and Limitations

As far as the authors know, this study is the first to use the B&H model to OV at the regional level in the EMR. An important strength of the study is the use of a feminist lens and analysis which centers women's voices and experiences at the core of improving RMC and QoMC. This study complements the various studies on QoMC that focused on a purely quantitative analysis of process, outcome, or output indicators, or qualitative analyses of determinants and barriers to maternal care.

However, this study is not without limitations. While an exhaustive literature search was conducted on mistreatment in childbirth, it is possible new publications and other relevant documents may have been missed. The limited literature and prevalence studies in the EMR hinder a comparative analysis intra- and inter-regionally as half the countries in the Region were not represented or mentioned in the literature. This review included literature from 1/6 HICs, 7/11 MICs, and 3/5 LICs (Tables 1, 2), possible publication bias may be negated by the generally equal distribution of publications among six countries (a quarter of the Region); however, only one study was included for each of Lebanon, Tunisia, and Yemen. The authors are cautious of the risk of ecological fallacy in generalizing these study data as overall prevalence across these countries. The small sample sizes of these studies inhibit conclusions to be made at national or regional levels as available data is fragmented and inconsistent as data on QoMC, RMC, D&A, OV is not collected homogenously or routinely at the facility, country, or regional level to allow sufficient estimation of prevalence and comparison. Additionally, publications related to QoMC are complex and heterogenic in nature, due to the variety of factors that influence patient satisfaction, especially during and following FBD. Re-call and courtesy/desirability bias may be present in the qualitative studies where patients fear criticizing providers or report higher-than-expected satisfaction overshadowed healthy maternal and child outcomes (62). Furthermore, inconsistencies in findings indicate steep under-reporting and bias in available data regarding OV in the Region and limited awareness of these human rights violations at individual, community, health system, and policy levels. While this framework is simple in its presentation, the list-format of the B&H model inhibits the conceptualization of the interconnectedness, compounding, and overlap of the types of abuses. The lack of standardized definitions of each type of D&A makes it even more difficult to compare systematically across studies, countries, and regions, particularly as various studies set their own definitions and sub-themes (6, 7, 19, 21, 27, 28, 82). While updated frameworks have expanded to explicitly capture sexual abuse, verbal abuse, legal, political, personal, health system, and socio-cultural factors affecting D&A, many still use list-format or more complex organization of themes (19, 21, 67, 68, 82). Finally, while the authors attempted to explain the intersectionality of the terms and concepts in Figure 2, this study is also limited by the controversies in conceptualizations (e.g., mistreatment vs. dissatisfaction vs. OV vs. D&A) and inconsistencies in terminologies in a nascent topic in the empirical literature (2, 6, 10, 19–23, 78, 90).

## Conclusions and Recommendations

Globally, over a third of women experience D&A in childbirth, and in the EMR, while an exact prevalence was difficult to capture, women's narratives indicate the normalization of OV in intrapartum care. To ensure that every birth in the EMR is respectful, rights, and evidence-based, multi-sectoral and multi-level actions are required to eliminate OV (19, 20, 82, 87–89). The proposed recommendations are based on the latest evidence and should be tailored to country-specific contexts to address women's needs and local challenges, within the context of health systems strengthening and national efforts to improve gender equality (2, 4, 8, 10, 11, 28, 29, 82, 87–89).

At the individual and community level, advocacy, education, and empowerment are required to push OV and women's rights in the childbirth agenda forward and to eliminate D&A. Partnerships with local women's empowerment champions, and civil society organizations may facilitate programming to address socio-cultural beliefs with regards to women's empowerment around decision-making, bodily autonomy, SRHR, gender, GBV, and power.

At facility and health systems levels, infrastructural and institutional cultural changes are needed to operationalize high-quality, respectful, women-centered rights-based care in the EMR. Introducing a multi-disciplinary team-based approach in intrapartum care, considering task-shifting and task-sharing care with nurses and midwives, and involving patients in the decision-making process may reduce elements of hierarchical care. In-service capacity building and mandating all maternal health workers receive cultural competency training on providing RMC, specifically on recognizing elements of D&A, understanding patient-provider power dynamics, and facilitating informed decision-making and consent is crucial to implementing women-centered care. Countries in the EMR would benefit from instituting midwifery schools in the region to prepare the necessary workforce to introduce and scale-up midwifery-led care which is more woman-friendly compared to the obstetric model of care. Further, integrating RMC and rights-based approaches to medical education is needed to sensitize health workers and improve evidence-based practices.

In addition to this, health facilities in the EMR may consider adopting and institutionalizing policies that improve quality, safety, efficiency, and patient-centeredness, while enabling a rights-based approach to birth free from violence and discrimination. EMR hospitals would benefit from implementing WHO's latest recommendations on improving intrapartum care including the following: providing woman-centered and quality childbirth education classes and resources, reducing routine interventions as standards of care (e.g. overused C-Sections, inductions, uterine pressure, vaginal exams and episiotomies, restricted food and water, and bed-confinement), allowing women to be mobile during labor and push in an upright position (or in their positions of choice), providing adequate pain relief options and alternatives during labor, and allowing choice for labor and birthing companions. Furthermore, investments in flexible and agile infrastructure, reconfiguring space in shared labor rooms to ensure women's privacy and limiting a number of students observing each birth, and obtaining women's consent should be of high priority.

Additionally, strengthening health information systems and referral pathways at the facility and national levels may reduce overcrowding, ameliorate patient flow, and ensure that women arrive at facilities when necessary instead of too-soon/too-late. The establishment of mechanisms to report abuses in childbirth, access to health workers and patients, regular monitoring by hospital managers, accreditation bodies, and quality-assurance boards are all recommended to improve accountability. Finally, integrating public health experts in health management teams and investment in public health research are essential.

Evidence-generation in all EMR countries is needed to measure national prevalence, compare patterns based on personal characteristics, settings, facility-types, geography, and measure progress over time and the health, social, and financial impacts of these RMC interventions. Qualitative data on QoMC would also be beneficial to capture the complexities and nuances between experiencing and reporting various experiences of D&A, mistreatment in childbirth, and OV. Further research is needed to document the experiences of women in childbirth, health workers, advocates, and other relevant stakeholders while evaluation studies are needed to further expose gaps in QoMC and knowledge-to-practice-translation of clinical guidelines.

At national and policy levels, advocacy and multi-stakeholder engagement are needed, along with strengthening accountability, governance, information systems, to ensure gender-sensitive policymaking and knowledge translation. Creation, monitoring, and evaluation of operational plans, RMC indicators, and monitoring mechanisms are needed to measure impact and improve QoMC at the facility and systems level.

In conclusion, OV threatens to provide women with dignified, respectful, rights-based, high-quality maternal care. It violates human rights through the provision of disrespectful and abusive care, reduces utilization and trust in health systems, and results in poorer health outcomes for women, children, families, and communities. Policymakers must prioritize the elimination of these human rights abuses and commit to eliminating OV.

## Author Contributions

MK is responsible for the conceptualization, data analysis, and first draft of this work for her master's thesis. KC supervised, supported the conceptualization, methodology, and interpretation of results. TK-K contributed to substantive revisions of the manuscript. All authors have read and approved the final manuscript.

## Conflict of Interest

The authors declare that the research was conducted in the absence of any commercial or financial relationships that could be construed as a potential conflict of interest.

## Publisher's Note

All claims expressed in this article are solely those of the authors and do not necessarily represent those of their affiliated organizations, or those of the publisher, the editors and the reviewers. Any product that may be evaluated in this article, or claim that may be made by its manufacturer, is not guaranteed or endorsed by the publisher.

## Supplementary Material

Supplementary Table 1 Search strategy.

Supplementary Table 2 Included articles by country, type of study, and category of Disrespect and Abuse (D&A).

Supplementary Table 3 Examples of D&A by theme and sub-theme in selected Eastern Mediterranean Region (EMR) countries from the included studies.

## Abbreviations

ANC: Ante-natal visit coverage
B&H: Bowser and Hill
D&A: Disrespect and Abuse in Childbirth
EMR: Eastern Mediterranean Region
FBD: facility-based deliveries
GBV: gender-based violence
L/M/HICs: low/middle/high-income countries
NGO: non-governmental organization
OV: obstetric violence
QoMC: quality of maternal care
RMC: respectful maternity care
SBA: skilled-birth attendant/ce
SDGs: Sustainable Development Goals
SRHR: Sexual and Reproductive Health and Rights
SSA: Sub-Saharan Africa
TLTL: Too Little Too Late
TMTS: Too Much Too Soon
UHC: Universal Health Coverage
WHO: World Health Organization.
