# Supplementary material for: Exposing Obstetric Violence in the Eastern Mediterranean Region: A Review of Women's Narratives of Disrespect and Abuse in Childbirth
Source: Front Glob Womens Health. 2022 Apr 25;3:850796. doi: 10.3389/fgwh.2022.850796 (PMC9082810; doi:10.3389/fgwh.2022.850796)
Supplement: Supplementary Table 1 — Search strategy. [file Data_Sheet_1.docx]

**Exposing Obstetric Violence in The Eastern Mediterranean Region:** A review of women’s narratives of Disrespect and Abuse in Childbirth

# **Annex 1:**

| Summary of Search Strategy by Publication Type and Objectives | | |
| --- | --- | --- |
| Search Strategy | **Details/Keywords** |  |
| Includes | Systematic reviews and peer-reviewed articles, grey literature, media and news articles, WHO/UNFPA/UN publications, and reports |  |
| Language | English |  |
| Time Frame | Only articles published after 2010 |  |
| Peer-Reviewed | | |
| PubMed | (((("disrespect and abuse"[All Fields] OR (obstetric[All Fields] AND ("violence"[MeSH Terms] OR "violence"[All Fields]))) OR (respectful[All Fields] AND ("mothers"[MeSH Terms] OR "mothers"[All Fields] OR "maternal"[All Fields]) AND care[All Fields])) OR ("parturition"[MeSH Terms] OR "delivery, obstetric"[MeSH Terms])) OR (mistreatment[All Fields] AND ("pregnant women"[MeSH Terms] OR ("pregnant"[All Fields] AND "women"[All Fields]) OR "pregnant women"[All Fields]))) OR "patient satisfaction"[MeSH Terms] AND ("middle east"[MeSH Terms] OR "africa, northern"[MeSH Terms] NOT "Turkey"[All Fields]) NOT "Israel"[All Fields] AND "2010/04/23"[PubDate] : "2020/04/19"[PubDate] |  |
| CINAHL | “respectful maternal care” OR “obstetric violence” OR “disrespect and abuse” OR “childbirth or labour or birth or labor or delivery” AND “perceptions or attitudes or opinion or experience”; (Published Date: 2010-2020, English Lang, Geographic Subset Middle East) |  |
| Cochrane Databases | "Respectful Maternal Care", "Obstetric Violence", "Disrespect and Abuse” |  |
| Eastern Mediterranean Health Journal (EMHJ) | "Respectful Maternal Care", "Obstetric Violence", "Disrespect and Abuse” |  |
| VU Libraries | “Respectful Maternal Care”, “Obstetric Violence”, “Disrespect and Abuse” [AND} “Middle East” OR “North Africa” (2010-2020; English Language) |  |
| Google Scholar | “obstetric violence”, “Disrespect and Abuse in Childbirth”, “Respectful Maternal Care”, “Dissatisfaction in intrapartum”; English after 2010 |  |
| Grey Literature | | |
| Google Scholar | “obstetric violence”, “Disrespect and Abuse in Childbirth”, “Respectful Maternal Care”, “Dissatisfaction in intrapartum” AND each of the 22 countries in EMR; Publications in English after 2010, removing duplicates) |  |
| Google | Keywords variations: RMC, OV, D&A, facility-based deliveries, quality of maternal care, mistreatment in childbirth, neglect, verbal or physical violence, dignity, rights-based, patient-centered, routine care, labor, delivery, C-sections, maternal health, pregnant women, women’s perceptions of deliveries, women’s expectations of deliveries, Eastern Mediterranean Region, Middle East, North Africa, Arab States, Fragile Conflict Settings, Humanitarian, Post-Conflict, emergencies |  |
|  | AND each of the 22 countries in the EMR |  |
| Academia | “Obstetric Violence” AND “Middle East North Africa” |  |
| Harvard Maternal Health Task Force (MHTF) | “Respectful Maternal Care” |  |
| Snowballing | Due to literature limitations, review of references of systematic reviews and global literature will identify other relevant literature, especially related to EM Region, low- and middle-income countries and developing countries. |  |

# **Annex 2:**

| Country | Type of Study | Author | Year | Methods | Sample size | Setting (hospital/facility-based or community), urban or rural, private or public, if known | 1. Physical abuse | 2. Non-consented care | 3. Non- confidential care | 4. Non-dignified care | 5. Discrimination | 6. Abandonment | 7. Detention | Other factors (socio-cultural, health system, or other) |
| --- | --- | --- | --- | --- | --- | --- | --- | --- | --- | --- | --- | --- | --- | --- |
| Afghanistan | Quali | Thommesen et al | 20 | Explorative case-study approach, participatory observation, in-depth interviews (n=14) and focus-group discussions (FGDs), (n=39) | 53 | Rural setting, community-level |  |  | **x** | **x** |  | **x** |  | **x** |
|  | Quali | Rahmani et al | 13 | One-to-one semi-structured interviews of 27 individuals, including 12 women who were pregnant or had recently given birth, seven doctors, five midwives, and three traditional birth attendants. The interviews were carried out in Kabul and the village of Ramak in Ghazni Province. | 27 | Both urban and rural settings, including community-level, not specifically limited to facility-based deliveries | **x** |  |  | **x** |  |  | **x** | **x** |
|  | Quali* | Currie et al | 19 | NGO Report (based on qualitative study, FGD, interviews, observations). FGD were conducted with new mothers. In-depth interviews were conducted with 20 policy makers and central and provincial officers from MoPH and other relevant stakeholders | FGD (n=64), IDI (n=20) | Public District hospitals outside provicial capitals: Balkh, Kandahar, Herat and Nangarhar. Using 2017 data from the National Health Management Information System, a list of public district hospitals outside of the provincial capital city was generated and one hospital per province randomly selected. Clients from the catchment area of the clinic were selected using the clinic register book for convenience | **x** |  |  |  |  | **x** |  | **x** |
|  | Quali | Arnold et al | 19 | Six weeks of observation, 41 background interviews, 23 semi-structured interviews with doctors, midwives and care assistants. Focus groups were held with two diverse groups of women in community settings | 23 | Community setting |  |  |  | **x** |  | **x** | **x** | **x** |
| Egypt | Commentary* | Khalil | 19 | N/A | N/A | N/A | **x** | **x** |  | **x** |  |  |  |  |
|  | Quanti | Elgazzar et al | 18 | Descriptive design using a survey tool (n=214) | 214 | Large public teaching hospital in urban city center (Mansoura city hospitals) |  | **x** |  | **x** |  | **x** |  | **x** |
|  | Quanti | Abdel Ghani eta l | 11 | Cross sectional study(n=400); each interviewed using a structured questionnaire | 400 | Major public teaching hospital in capital city, Cairo (El Kasr- Aini, - Cairo- University Maternity Hospitals) | **x** |  | **x** | **x** |  | **x** |  | **x** |
|  | Quanti | Mousa et al | 18 | Cross-sectional retrospective study, purposeful sampling, using structured questionnaire as survey tool (n= 501) | 501 | Large public teaching hospital in urban city center (Minia University Maternity and Child Health Hospital in Minia, Egypt.) | **x** |  |  | **x** | **x** | **x** |  |  |
|  | Quanti | Monazea et al | 15 | Cross-sectional, (n=435) interviewed using a semistructured questionnaire | 435 | Large public teaching hospital in major urban city center (Assiut University Hospital) |  | **x** | **x** | **x** |  | **x** |  | **x** |
| Iran | Quali | Mohammadi et al | 17 | In-depth interviews were conducted with 11 participants who were prospectively recruited (n=11) | 11 | One university hospitals in capital, Tehran |  |  |  | **x** | **x** |  |  | **x** |
|  | Quali | Fathi Najafi et al | 17 | Exploratory and qualitative study, observations and semi-structured interviews with health workers, birth companions and new mothers (n=25)recruited through a purposive sampling method.   A total of 25 women, including 16 women in labor and having the experience of a natural childbirth, two women who had recently given birth, one birth companion, one doula midwife, two midwives, one obstetrician,one resident in obstetrics, and one medical student, were finally selected. The husband of one of the participants, as well as a midwifery student and lecturer in midwifery were also included. | 25 | Mostly hospitals in major city of Mashhad  Participants were selected from hospitals and health centers, The hospitals included four governmental hospitals, one social health-care hospital, three non-governmental (private) hospitals and two charity hospitals, members of websites, public places such as parks, and social networks like Telegram and Viber (by sending an invitation letter and an information sheet). |  |  |  |  |  | **x** |  |  |
|  | Quali | Pazandeh et al | 17 | Descriptive qualitative study, purposeful sampling, interviews (n=26) | 26 | Four public hospitals in capital city, Tehran with a high rate of births, providing services to low and middle income families. | **x** | **x** |  |  |  |  |  |  |
|  | Quanti | Ghanbari-Homayi et al | 19 | Cross-sectional study, cluster sampling (n=800), structured questionnaire used; Data were collected through face to face interviews and analysed mainly by multivariable logistic regression. | 800 | 64 health centres (public) in Tabriz, the second largest city in Iran. | **x** | **x** |  |  |  |  |  | **x** |
|  | Quanti | Tabrizi et al | 14 | Cross-sectional study (n=200) using survey tool | 200 | One large teaching hopsital in major city, Tabriz |  |  | **x** | **x** |  |  |  | **x** |
| Iraq | MM | Shabila et al | 15 | Mixed Methods, using Q-methodology questionnaire (n=37) purposively selected to increase sample diversity; 2 focus group with 20 women each, 5 indepth-interviews and 3 health workers (2 nurses and 1 gynocologist) | 37 | Public facility in Erbil, capital of Iraqi Kurdistan Region | **x** |  | **x** | **x** |  | **x** |  | **x** |
|  | Quanti | Atiya et al | 16 | Purposive sample of (200) postpartum women, structured questionnaire and interviews | 200 | Large teaching hospital in capital, Baghdad |  |  |  | **x** |  |  |  | **x** |
|  | Quanti | Ahmed et al | 20 | Cross-sectional study was conducted on a convenient sample of 1196 women who were directly interviewed using a structured questionnaire | 1196 | Erbil City (urban) and recruited from: Maternity Teaching Hospital is the biggest specialized public hospital for women’s Health care especially labor and delivery care with 300 hundred beds and 30 beds in delivery room. Rezgary Hospital is a general hospital including a unit for women’s health care services with capacity of 6 beds and Malafandy Primary Health Center has a small unit with capacity of 5 beds for labor and delivery care of women with low risk and normal health conditions. All of these institutions are **public** health settings. |  | **x** | **x** | **x** |  |  |  | **x** |
|  | Quanti | Asadi et al | 18 | Descriptive cross-sectional study using a structured questionnaire (n=459) | 459 | 14 primary health care centers (public, urban) | **x** | **x** | **x** | **x** |  | **x** | **x** | **x** |
| Jordan | MM | Shaban et al | 11 | Explorative research design with non-participant observation (n=460), proportional stratified sample was recruited | 460 | Three major public hospitals in Jordan | **x** | **x** | **x** |  |  | **x** |  | **x** |
|  | MM | Mohammad et al | 14 | Descriptive cross-sectional study, semi-structured interviews using questionnaire (n = 320) with women immediatel post-partum (2-24hrs after birth); details of recruitment not specified | 320 | Three major public hospitals in Jordan |  | **x** | **x** |  |  |  |  | **x** |
|  | Quali | Khresheh et al | 19 | Exploratory and qualitative design utilizing semi-structured interviews (n=21) | 21 | The main public hospital in Southern Jordan, serving the entire southern region |  |  | **x** | **x** |  | **x** |  | **x** |
|  | Quali | Hatamleh et al | 13 | Semistructured interviews (n= 460) | 460 | Three public hospitals in Jordan | **x** |  | **x** | **x** |  | **x** |  | **x** |
|  | Quanti | Alzyoud et al | 18 | Retrospective cross-sectional descriptive design, using structured questionnaire/survey tool (n=390) | 390 | 4 governmental Maternal and Child Health Centers (MCHCs) in Zarqa, Jordan selected due to their size and diversity (urban, public, facility-based) | **x** | **x** |  | **x** | **x** | **x** |  | **x** |
| Lebanon | Quali | Kabakian-Khasholi et al | 13 | Inductive qualitative design, using face-to-face semi-structured interviews (n=22) | 22 | Recruited through perinatal database and selected obstetricians' clinics in the Greater Beirut area in Lebanon (facility-based, urban, most likely private facilities due to contract out mechanisms in Lebanon) | **x** | **x** |  |  |  |  |  | **x** |
| Multi-country (MC): Egypt, Lebanon, Palestine and Syria | Review | Kabakian-Khasholi et al | 12 | Literature Review | N/A | N/A | **x** | **x** |  |  |  |  |  |  |
| MC: Egypt, Lebanon, Syria | MM | Kabakian-Khasholian et al | 17 | Questionnaire (n =2620), medical charts also reviewed | 2620 | Three major public teaching hospitals in major cities each country |  | **x** | **x** | **x** |  |  |  | **x** |
| MC: United Arab Emirates, Jordan, Egypt, Qatar, Lebanon and Saudi Arabia. | Review | Hussein et al. | 17 | Narrative Review | N/A | N/A | **x** | **x** | **x** | **x** |  | **x** |  | **x** |
| Pakistan | Quali | Askari et al | 14 | purposeful approach in-depth interviews (n=21) | 21 | Gonabad University of medical sciences (tertiary teaching hospital, public and urban) | **x** | **x** | **x** |  |  |  |  |  |
|  | Quanti | Azhar et al | 18 | Cross sectional household based study, interview based structured questionnaire (n = 360). | 360 | Household based study was conducted in tehsil Kharian of district Gujrat. (Community-level, semi-urban, public facility) | **x** | **x** | **x** | **x** | **x** | **x** | **x** | **x** |
| Saudi Arabia | MM | Altaweli et al | 19 | Mixed-methods: exploratory study using an ethnographic approach. Data collection methods included participant observations of 19 labors and births (n = 8 at City Hospital and n = 11 at King's Hospital) and semi‐structured interviews with 29 health care professionals. | Obs (n=19), IDI (n=29) | Two public teaching hospitals in Jeddah (major cities) | **x** | **x** | **x** | **x** |  |  |  | **x** |
|  | Quali | Scamell et al | 17 | Ethnographic data collection methods, including participant observation, field notes, and interviews of patients (n=19) and health workers: obstetricians (n=10), midwives (n=12), obstetric nurses (n=6) and nurse/midwives (n = 1). Health workers assisted in recruiting the sample participants. | 19 | Public hospital in major city, Jeddah | **x** |  | **x** | **x** | **x** | **x** |  |  |
|  | Quali | Jahlan et al | 16 | Cross-sectional/qualitative IDI (n=32) and questionnaire (n=169) 24 hours post-partum in 3 selected public hospitals | 201 | Three public hospitals in major cities | **x** | **x** | **x** | **x** |  | **x** |  | **x** |
|  | Quanti | Alnemari | 20 | Cross-sectional study (n=358), recruited after thorough non-probability consecutive sampling technique, using structured questionnaire | 358 | (Tertiary hospitals, urban, public)  Post-natal clinics of maternity hospital of King Faisal medical complex and Al-Hada armed forces hospital, Taif city, Saudi Arabia. | **x** | **x** | **x** | **x** |  | **x** | **x** | **x** |
|  | Quanti | Al-Mandeel et al | 13 | Prospective cohort study (n= 402) using a structured standardized translated questionnaire with fixed-choice questions | 402 | Three governmental tertiary hospitals within capital city, Riyadh, including a university hospital (King Khalid University Hospital, KKUH), a Ministry of Health Hospital (King Fahd Medical City, KFMC), and a military hospital (Riyadh Military Hospital, RMH). |  |  |  |  |  | **x** |  | **x** |
| Sudan | Quanti | Handady et al | 15 | Cross sectional (n=284), hospital based study, participants selected through systematic random sampling and structured questionnaire | 284 | One large teaching hopsital in major city, Omdurman (Omdurman Maternity Hospital) | **x** | **x** | **x** | **x** |  |  |  | **x** |
|  | Quanti | Altahir et al | 18 | Descriptive cross-sectional study in 3 hospitals proportionally sampled based on the number of clients who received childbirth services at each facility, semi-structured interviews (n= 263) using structured survey tool | 263 | Three public teaching hopsitals in the capital and urban city-centers. Omdurman Maternity Hospital, Saad Abu-Aleila teaching hospital and Khartoum north teaching hospital were randomly selected for this study. | **x** | **x** | **x** | **x** | **x** | **x** | **x** | **x** |
| Tunisia | Quali | Amroussia et al | 17 | Qualitative IDI (n=11) recruited voluntarily through two local NGOs | 11 | Public tertiary hospitals (urban): Seven participants delivered at a University Teaching Hospital in the capital; while the other participants delivered at public healthcare facilities in different cities (3 different regional hospitals and one university teaching hospital in a coastal city). | **x** | **x** |  | **x** | **x** |  |  | **x** |
| Yemen | MM* | Kempe | 19 | PhD Thesis: multi-stage (stratified-purposive-random) sampling(n=220), semi structured questionnaire | 220 | Both urban and rural settings, including community-level, not specifically limited to facility-based deliveries | **x** | **x** |  | **x** |  | **x** |  | **x** |
|  | *Not peer-reviewed article | | | | | | | | | | | | | |

# **Annex 3:**

| Country |  | Egypt | | | | Iraq | | Jordan | | | Pakistan | Saudi Arabia | | Sudan | |
| --- | --- | --- | --- | --- | --- | --- | --- | --- | --- | --- | --- | --- | --- | --- | --- |
| Author |  | **Elgazzar et al** | **Abdel Ghani et al** | **Mousa et al** | **Monazea et al** | **Ahmed et al** | **Asadi et al** | **Shaban et al** | **Mohammad et al** | **Alzyoud et al** | **Azhar et al** | **Alnemari** | **Al-Mandeel et al** | **Handady et al** | **Altahir et al** |
| Sample size/ D&A Theme | D&A Sub-theme | **214** | **400** | **501** | **435** | **1196** | **459** | **460** | **320** | **390** | **360** | **358** | **402** | **284** | **263** |
| 1. Physical abuse | *General* |  |  |  |  |  | 6.5% exposed to physical violence |  |  |  |  | 18.6% experienced (0.6% Reported) |  |  | 8.3% reported physcial abuse |
|  | Overuse of routine interventions |  | x | x |  |  | Bed confinement: 51.6% of surveyed laboring women were confined to the bed Fundal Pressure in Labor: 36.2% | Induction: 95% of all women having their labor augmented as a routine policy Episiotomy: More than one third (37%) of study participants had an episiotomy with varying degrees of laceration (96%) Forced lithotomy position: 100% of women were routinely placed in lithotomy position for labor and delivery Frequent vaginal exam: The majority of women (89%) had frequent vaginal examinations, which were defined as being carried out less than once every 4 hours Continuous and restrictive blood pressure and fetal monitoring : 77% | Frequent vaginal exam:45% of respondents receiving more than 8 vaginal exams Induction: approximately 95% of all women having their labor augmented as a routine policy Episiotomy: 37% (both primiparous and multiparous) experienced episiotomy.  Being in lithotomy position during birth: 79.4% | x | x | Frequent and forceful vaginal exam: 25% of participants reported painful vaginal examinations Frequent vaginal exams: 25%,  Pushing on abdomen forcefully in labor: 21.8%,  Denial of food and drink and routine use of IV fluids: 11.2% Bed confinement: 2.5% participants responded that staff members used any kind of mouth muzzle or restricted them in bed during childbirth. |  | Induction: 73.2% of participants reported being artificially augmented/induced Episiotomy: 94.7% of study-participants reported being routinely subjected to episiotomies Artificial rupture of membrane: 85% Forced labor position: 83%  Routine IV fluids: 60% |  |
|  | Hitting |  |  | 15.6% hit by health worker |  |  |  |  |  |  |  | 4.5% reported hitting, slapping, pushing and/or pinching by staff during childbirth |  |  |  |
|  | Insufficient pain medication |  |  |  |  |  | 76% of survey-respondents were dissatisfied by the availability and 35% dissatisfied by the strength of pain medications |  |  | 34% reported not receiving pain medication upon request during labor 28% reported having episiotomies stiched without anesthesia |  | 13.4% operated on without waiting for effects of anesthesia to settle |  |  |  |
|  |  |  |  |  |  |  |  |  |  |  |  |  |  |  |  |
| 2. Non-consented care | *General* |  |  |  |  |  |  |  |  |  | **Experienced by 97.5 % vs. 11.1% reported** |  |  |  |  |
|  | **Hierarchical care and limited decision-making power** | **100% of survey respondents reported dissatisfaction with their involvement in decision-making** |  |  | **x** | **x** | **48.3% of survey respondents reported not being involved in decisions of their injections** | **x** | **x** | **45% reported no participation in clinical decision-making (e.g., receiving pain control, episiotomy, and oxytocin),** |  | **x** |  | **57% of survey respondents reported not being involved in decision-making** | **x** |
|  | **Limited information for decision-making and consent** |  |  |  | **88% reported being deprived of information and updates related to labor** | **45.7% were not satisfied with how the provider explained the examination/assessment, 47.9% not satisfied with how provider explained health problem/diagnosis, 42.4% dissatisfied with the treatment options/choices offered** |  | **70% not given infomraiotn about labor outcomes and progress** |  | **80% reported being deprived of information and updates related to labor** |  |  |  | **65.8% did not have doctors introduce themselves or their roles in care** | **77.4 % reported being deprived of information and updates related to labor** |
|  | **Unconsented routine interventions** |  |  |  |  |  |  |  |  |  |  | **19% of women underwent episiotomies, 15% forced to deliver in non-preferred position, 3.6% C-sections, 1.4% tubal ligations and 0.3% hysterectomies, routinely administered without consent** |  | **21.8% were asked permission or consent before being examined or delivering any procedure** | **35% were subjected to unconsented procedures** |
|  |  |  |  |  |  |  |  |  |  |  |  |  |  |  |  |
| 3. Non- confidential care | *(General lack of privacy)* |  | **86.5% reported the need for maintaining privacy in all procedures** |  | **37% were dissatisfied with confidentiality in care; only 17.9% were fully satisfied with privacy in care** | **44% of surveyed women were not satisfied with privacy during medical exams** | **64.3% of respondents felt their privacy was not preserved during birth** | **67% of surveyed women reported lack of privacy** | **x** |  | **58.6% of surveyed women reported lack of privacy (4.2% reported it)** | **x** |  | **19 % of surveyed women reported lack of privacy** |  |
|  | **Lack of physical protection of patient confidentiality** |  |  |  |  |  |  |  |  |  |  | **Reported that staff members discussed their private health information in public (11.5%) and to others/ relatives (10.1%).  11.2% whose body was seen by others (non-staff members) during child birth and 5.6% were in the delivery room without curtains between beds.** |  |  | **32.3% reported providers did not use curtains or visual barriers to protect mothers' privacy during childbirth; 16.3% of mothers reported that their files are not stored in cabinets with limited access.** |
|  | **Overcrowding** |  |  |  |  |  |  | **60% of respondents dissatisfied with privacy due to having to share a room with other laboring women** |  |  |  |  |  |  |  |
|  |  |  |  |  |  |  |  |  |  |  |  |  |  |  |  |
| 4. Non-dignified care | *General* | **x** | **x** | **x** | **x** | **x** | **x** |  |  | **x** | **45.6% experienced (12.2% reported)** | **x** |  | **x** | **x** |
|  | **Verbal abuse** |  |  | **45.9% postpartum mothers reported being yelled at by healthcare workers because they have not done what they were told to do during childbirth.** |  | **More than half of the study sample were not satisfied with this verbal communication: “introduce self” (66.5%), “greeted the patient” (61.7%), “checked the patient’s understanding” (58%), “asked whether patient had other issues or concerns” (50.2%).** | **10.1% reported being exposed to verbal violence** |  |  | **40% of the women reported having experienced at least one form of verbal abuse during their last birth experience: 17.6% (n=69) reported that the health care team used aggressive tones of voice during their birthing experience.   22% of participants reported being ridiculed by the health care team at their last birth and 19.4% (n=76) were spoken to harshly or sworn at.  About 20 % of participants reported being verbally threatened** |  | **21% reported being scolded and 20% threatened** |  |  | **58% reported being insulted and 28% verbally abused** |
|  | **Dehumanized care** | **37.4% and 25.2% were dissatisfied and uncertain about their psychological care; due to poor emotional support from nurses during intrapartum care.** | **67.5% of them reported there is a need for qualified nurses offer help and demonstrate empathy. 57.5% prirotized availability to ventilate and expressing fear and anxiety without shame** | **Less than ﬁfty percent of postpartum mothers felt that the health workers were concerned about them, were empathic and treated them with respect as individuals.** | **60.9% were dissatisfied with the interpersonal aspects of care** | **27% were dissatisfied with providers maintaining good eye contact, 26.1% dissatisfied with provider using attentive/caring body language, 25.6% found provider did not use a professional tone of voice to convey empathy, 26.3% found providers did not listen carefully and interrupted patient while speaking, 28.2% dissatisfied with provider non-verbal cues, 27.8% dissatisfied with provider posture and facial expressions** | **45.5% did not feel recieved respectfully,** |  | **31% of women reported that they did not experience politeness, courtesy, or respect from care providers and did not have an opportunity to clarify advice or information.   22% of women provided descriptive comments about being treated as if they were a machine with no sense of individualized care and a lack of encouragement during labor and birth.** |  |  | **6.4% reported being treated by a staff member as a passive participant during child birth** |  |  |  |
|  |  |  |  |  |  |  |  |  |  |  |  |  |  |  |  |
| 5. Discrimination | *General* |  |  |  |  |  |  |  |  | **x** | **23.6% experienced (5.3% reported)** |  |  |  | **x** |
|  | **Personal characteristics** |  |  | **86.82% of postpartum mothers felt that healthcare workers mistreated them because of some personal characteristics, 85.1% felt health workers offended them or their companions based on their personal characteristics.** |  |  |  |  |  |  |  |  |  |  | **6.1% of the participant reported discrimination mainly due to their socio-economic status (33%).** |
|  | **Language** |  |  | **61.48% that healthcare workers communicated to them in a language outside their level of understanding** |  |  |  |  |  |  |  |  |  |  | **2% of respondents reported the use of unclear or difficult language by providers.** |
|  |  |  |  |  |  |  |  |  |  |  |  |  |  |  |  |
| 6. Abandonment | *General* | **x** | **x** | **x** | **x** |  | **x** | **x** |  | **x** | **72.5% experienced (11.1% reported)** | **x** | **x** |  | **x** |
|  | **Lack of companionship** |  |  |  | **95% of participants did not have a companion, 37.2% were dissatisfied with this.** |  | **65.1% dissatisfied that accompanying person could not stay with her,** | **99% (Only 1% of the women were able to have a support person or companion during labour and birth).** | **75% of women made specific comment about the lack of human support from midwives or doctors during their labor, including: non-health care staff, any form of female support, a female friend or family member.** |  |  | **47% of the study participants responded that staff members did not allow the presence of any relative during child birth.** |  |  |  |
|  | **Neglect** | **56.1% and 16.8% were uncertain and dissatisfied with continuity of care; likely due to nurses leaving women for a long time during the first and fourth stages of labor** | **67.5% of participants indicated the need for quick response to request, 52.8% for frequent monitoring and 47.2% for accessibility of caring medical staff** | **59.68% stated that they were not given prompt service and waiting time is long.** |  |  | **Insufficient and abandoned care: 44.5% did not receive assistance in labor, 27% not examined postpartum (mom and baby), 22% fetal heart rate not checked** |  |  | **Unempathetic Care: 11% of participants reported the main reason for abandonment was providers “talking to each other” Insufficient care: 21.2% were dissatisfied due to  refusal by health professionals to assist them during or after giving birth Delivery alone: 30% of survey participants reported delivering without medical provider present** |  | **Delivery alone: 11% of respondents reported delivering without medical provider present Untimely Care: 26.3% of women had to wait a long time before getting medical care by staff members Insufficient Care: 18.4% faced ignorance for assistance requests from staff members during childbirth** |  |  | **21.7% of mothers reported being left without attention during labor. (46.8% of described this delay as being reasonable, however the other 38.7% said it wasn’t reasonable and they couldn’t tolerate it at all.)** |
|  |  |  |  |  |  |  |  |  |  |  |  |  |  |  |  |
| 7. Detention | *General/Subtheme: Culture of bribes and Informal payments* |  |  |  |  |  | **x** |  |  |  | **x** | **Inappropriate demands for payments, bribes/informal pay requested from 2.5%   1.4% women reported that the baby was held at the facility due to failure to pay after child birth** |  |  | **1.9% of participants experienced detention due to financial reasons** |
